# Data from multi year and multi radiation detector measurements of different radiation sources with environmental data

**DOI:** 10.1016/j.dib.2020.105828

**Published:** 2020-06-07

**Authors:** Braden Goddard, George W. Hitt, Dorian Bridi, Abdel F. Isakovic, Reyad El-Khazali, Ayman Abulail

**Affiliations:** a Department of Mechanical and Nuclear Engineering, Virginia Commonwealth University, 401 West Main Street, Richmond, VA 23284, United States; b Department of Physics and Engineering Science, Costal Carolina University, 109 Chanticleer Drive East, Conway, SC 29526, United States; c Independent consultant, Vienna, Austria; d Department of Physics, Khalifa University, Abu Dhabi, UAE; e Department of Electrical Engineering and Computer Science, Khalifa University, Abu Dhabi, UAE

**Keywords:** Radiation, half-life, Decay constant, Radioactive decay, Continuous measurement, Decay fluctuations, Detector stability

## Abstract

The data described consists of radiation measurement outputs from several different detectors located in Abu Dhabi, UAE, for the duration of 30 November 2014 through 13 December 2016. This data could be useful for anyone studying radiation count rate or spectroscopic changes over a multi year period from various radiation sources and measurement systems. The outputs of the measurement systems include 1) counts per 20 min from a Geiger–Muller detector measuring ^90^Sr, 2) counts per 20 min from a Geiger–Muller detector measuring ^204^Tl, 3) radiation spectra per 30 min from a NaI detector measuring ^60^Co, 4) radiation spectra per 30 min from a NaI detector measuring ^54^Mn, 5) radiation spectra per 30 min from a liquid scintillation detector measuring ^226^Ra, 6) counts per 30 min from a liquid scintillation detector measuring ^14^C, and room pressure, temperature, and humidity data every 10 min. A detailed description of the setup of each of these measurement systems can be found in “Experimental Setup and Commissioning Baseline Study in Search of Time-Variations in Beta-Decay Half-Lives” [1].

**Specifications table**

**Table:** 

**Subject**	Physics and Astronomy: Radiation
**Specific subject area**	Radiation measurements and instrument stability
**Type of data**	Table
**How data were acquired**	Radiation measurement systems and environmental data logger.Instruments: NATS-1510/1520 g Geiger–Muller tube connected to a NATS-EDUC-1510 single channel analyzer (SCA) [2]. The software used was NATS Geiger–Muller counting software.Instruments: Canberra NAIS 2 × 2 NaI(Tl) LED temperature stabilized scintillation counter [3] connected to a Canberra Osprey multi-channel analyser [4]. Shielded with a with Canberra model 727 lead shield [5]. running Canberra's Genie 2000 Basic Spectroscopy software [6]. Data was extracted from the proprietary file format using Canberra's CAMTools [7].Instruments: ORDELA PERALS 8100AB-HV liquid scintillation counter [8] powered by an ORTEC 4006 minibin [9] connected to a NATS ARIA MCA [10]. The software was that of NATS's ARIA MCA.Instruments: Hidex Triathler 425–034 liquid scintillation liquid scintillation counter [11] running the Hidex Triathler software.Instruments: Madgetech PRHTemp101A environmental data logger running Madgetech software [12].
**Data format**	RawCSVASCII
**Parameters for data collection**	Different shielding, voltage, geometry, and background radiation conditions were considered before data was collected.
**Description of data collection**	Data was collected using commercially available radiation measurement systems and software.
**Data source location**	Institution: Khalifa UniversityCity/Town/Region: Abu DhabiCountry: UAEKhalifa University, L building, third floor GPS coordinates 24.4476, 54.3943samples/data: 30 November 2014 through 13 December 2016, local United Arab Emirates time zone.
**Data accessibility**	Repository name: Mendeley DataData identification number: doi: 10.17632/tfcc3jk869.1 Direct URL to data: http://dx.doi.org/10.17632/tfcc3jk869.1
**Related research article**	B. Goddard, G.W. Hitt, A.A. Solodov, D. Bridi, A.F. Isakovic, R. El-Khazali, and A. Abulail, “Experimental Setup and Commissioning Baseline Study in Search of Time-Variations in Beta-Decay Half-Lives,” Nuclear Instruments and Methods in Physics Research A, 812, pp. 60–67, doi.org/10.1016/j.nima.2015.12.026, 2016

## 1 Value of the data

The data presented consists of simultaneously acquired radiation counts from six different systems, three different detector types, and six different radioactive sources, along with environmental data. A multi year data set of this type does not publicly exist.
Anyone doing research with radiation measurement systems over a prolonged multi year period could benefit from this data. Examples of this data being applied to detector instability can be found in references [13,14].
This raw data can be analysed to gain insights into how temperature, pressure, humidity, and time of day/year affect radiation measurements. The fact that all six detector systems operated simultaneously in the same room allows for cross validation of any analyses.
This data has additional value because of the latitude and location it was collected. Very little radiation measurement data has been made publicly available from the United Arab Emirates.

## 2 Data description

The Geiger–Muller ^90^Sr data set consists of 20,305 data points, each taken approximately 20 min apart. The file name is “GM_Sr-90_29Mar2015to7Jan2016.csv” and can be directly accessed at https://data.mendeley.com/datasets/tfcc3jk869/1#file-5916cdc3-7e52-466a-87bb-574af6b4001d. The first row of this file contains header information “Acq. Data, Acq. Time stop, Total counts”. The first column is the acquisition date of each measurement in mm/dd/yyyy format. The second column is the acquisition time of each measurement in hh:mm:ss format. The third column is the number of counts recorded by the detector system during the 20 min measurement.

The Geiger–Muller ^204^Tl data set consists of 31,114 data points, each taken approximately 20 min apart. The file name is “GM_Tl-204_29Mar2015to27June2016.csv” and can be directly accessed at https://data.mendeley.com/datasets/tfcc3jk869/1#file-1a65a10b-5111-445e-ad77-3d9767b93215. The first row of this file contains header information “Acq. Data, Acq. Time stop, Total counts”. The first column is the acquisition date of each measurement in mm/dd/yyyy format. The second column is the acquisition time of each measurement in hh:mm:ss format. The third column is the number of counts recorded by the detector system during the 20 min measurement.

The NaI ^54^Mn data set consists of 22,686 spectroscopic measurements, each with 2048 channels. These measurements are taken approximately 30 min apart. The file name is “NaI_Mn-54_30Nov2014to1May2016.csv” and can be directly accessed at https://data.mendeley.com/datasets/tfcc3jk869/1#file-e4c8d98b-504a-4407-98c2-e5502e90706d. The first row of this file contains header information “Acq Date, Acq Time, LiveTime, RealTime, DeadTime, Slope, Offset, Integral, NoOfChannels”. The first column is the acquisition date of each measurement in mm/dd/yyyy format. The second column is the acquisition time of each measurement in hh:mm:ss format. The third column is the live time of the measurement, exactly 1800 s (30 min). The fourth column is the real time of the measurement. This is the real amount of time that the detector was measuring radiation for this measurement. The fifth column is the dead time in units of percent. This is calculated from the live time and real time and represents the amount of time that the detector was not able to detect radiation due to detector dead time. The sixth column is the slope of the energy calibration used to relate channel number to energy in keV. The seventh column is the offset of the energy calibration used to relate channel number to energy in keV. The energy calibration used is *E* = 1.531862*Ch-11.2678, where E is energy in keV and Ch is the channel number. The eight column is the sum of all the counts in all 2048 channels for that measurement. The ninth column is the number of channels used in the measurement (2048). The next 2048 columns are the number of counts in that particular channel.

The NaI ^60^Co data set consists of 25,780 spectroscopic measurements, each with 2048 channels. These measurements are taken approximately 30 min apart. The file name is “NaI_Co-60_1Dec2014to1June2016.csv” and can be directly accessed at https://data.mendeley.com/datasets/tfcc3jk869/1#file-0e2c316a-024c-40ba-af82-30297443121e. The first row of this file contains header information “Acq Date, Acq Time, LiveTime, RealTime, DeadTime, Slope, Offset, Integral, NoOfChannels”. The first column is the acquisition date of each measurement in mm/dd/yyyy format. The second column is the acquisition time of each measurement in hh:mm:ss format. The third column is the live time of the measurement, exactly 1800 s (30 min). The fourth column is the real time of the measurement. This is the real amount of time that the detector was measuring radiation for this measurement. The fifth column is the dead time in units of percent. This is calculated from the live time and real time and represents the amount of time that the detector was not able to detect radiation due to detector dead time. The sixth column is the slope of the energy calibration used to relate channel number to energy in keV. The seventh column is the offset of the energy calibration used to relate channel number to energy in keV. The energy calibration used is *E* = 1.536559*Ch-13.24, where E is energy in keV and Ch is the channel number. The eight column is the sum of all the counts in all 2048 channels for that measurement. The ninth column is the number of channels used in the measurement (2048). The next 2048 columns are the number of counts in that particular channel.

The PERALS ^226^Ra liquid scintillation data set consists of 30,709 timing spectroscopic measurements, each with 4096 channels. These measurements are taken approximately 30 min apart. The file name is “Perals_Ra-226_8Jan2015to13Dec2016.csv” and can be directly accessed at https://data.mendeley.com/datasets/tfcc3jk869/1#file-9af610e6-f22e-4b78-9546-0adbb952655d. The first row of this file contains header information “Acq Date, Acq Time, RealTime, DeadTime, Beta Gamma Counts, Alpha Counts, Integral, NoOfChannels”. The first column is the acquisition date of each measurement in mm/dd/yyyy format. The second column is the acquisition time of each measurement in hh:mm:ss format. The third column is the real time of the measurement, exactly 1800 s (30 min). The fourth column is the dead time in units of percent. This is calculated from the live time and real time and represents the amount of time that the detector was not able to detect radiation due to detector dead time. The fifth column is the estimated number of counts due to beta and gamma radiation. The sixth column is the estimated number of counts due to alpha radiation. The seventh column is the sum of all the counts in all 4096 channels for that measurement. The eight column is the number of channels used in the measurement (4096). The next 4096 columns are the number of counts in that particular channel.

The Triatheler ^14^C liquid scintillation data set consists of 30,012 data points, each taken approximately 30 min apart. The file name is “Triatheler_C-14_11Dec2014to13Dec2016.csv” and can be directly accessed at https://data.mendeley.com/datasets/tfcc3jk869/1#file-821bfd50-4e66-4de3-8c1f-538ceae3e356. The first row of this file contains header information “Acq. Data, Acq. Time stop, Total counts”. The first column is the acquisition date of each measurement in mm/dd/yyyy format. The second column is the acquisition time of each measurement in hh:mm:ss format. The third column is the number of counts recorded by the detector system during the 30 min measurement.

PRHTemp101A environmental data set consists of 85,695 data points, each taken approximately 10 min apart. The file name is “envirodata_10Feb2015to13Dec2016.csv” and can be directly accessed at https://data.mendeley.com/datasets/tfcc3jk869/1#file-5ed5caf1-765c-43e1-8acf-1c05804f05e2. The first six rows and four columns contain information about the PRHTemp101A device. The seventh row of this file contains header information “Date, Time, Temperature (C), Humidity (% RH), Absolute Pressure (mbar)”. The first column (starting from row eight) is the acquisition date of each measurement in mm/dd/yyyy format. The second column (starting from row eight) is the acquisition time of each measurement in hh:mm:ss format. The third column (starting from row eight) is the temperature in units of Celsius recorded by the environmental data logger during the 10 min measurement. The fourth column (starting from row eight) is the relative humidity in units of percent recorded by the environmental data logger during the 10 min measurement. The fifth column (starting from row eight) is the absolute pressure in units of mbar recorded by the environmental data logger during the 10 min measurement.

## 3 Experimental design, materials, and methods

The initial setup of the experimental measurement campaign can be found in detail in “Experimental Setup and Commissioning Baseline Study in Search of Time-Variations in Beta-Decay Half-Lives” [1]. What is not described in the above article are changes and disruptions to the data streams that occurred during the collection process. These changes and disruptions are described below.

The Geiger–Muller ^90^Sr data set periodically has time durations between measurements more than 20 min. This increased duration was caused by the manual nature of stopping the counting process while the data was saved. There is also a period of time (December 27 though 29, 2015) in which no data was recorded. This loss of data was caused by a power outage.

The Geiger–Muller ^204^Tl data set periodically has time durations between measurements more than 20 min. This increased duration was caused by the manual nature of stopping the counting process while the data was saved. There is also a period of time (December 27 through 29, 2015), (January 19 through 20, 2016), and (February 1 through 17, 2016) in which no data was recorded. This loss of data was caused by a power outage.

The NaI ^54^Mn data set has a discontinuity on May 3, 2015, at 10:49:18. This was caused by the flat top (FT) value unintentionally being changed from 1.0 µs to 0.8 µs.

The NaI ^60^Co data set has no noteworthy anomalies during the data collection.

The PERALS ^226^Ra liquid scintillation data set from January 8 through February 1, 2015 (Block 1) is different from the other data points. After acquiring this first data set, the fine and course gain were adjusted to move the peaks of interest towards the middle of the multichannel analyser window. The pulse shape discriminator value was adjusted to improve discrimination between the alpha and beta plus gamma peaks. Lead shielding was placed around the detector system to minimize influences from external radiation. The PERALS data set is missing data from (December 27 through January 5, 2016), (January 19 through 20, 2016), and (February 16 through 17, 2016). This loss of data was caused by a power outage.

The Triatheler ^14^C liquid scintillation data set from December 11, 2014 through February 18, 2015 (Block 1) is different from the other data points. Lead shielding was placed around the detector system to minimize influences from external radiation. From February 1 through 18, 2015 the Triatheler was used for a different experiment. The sample, detector system, and all settings were reset to their previous value before February 1, 2015. The Triatheler data set is missing data from (December 27 through January 5, 2016), (January 19 through 20, 2016), (February 13 through 17, 2016), (August 5 through 9, 2016), and (November 6 through 17, 2016). This loss of data was caused by a power outage.

PRHTemp101A environmental data set is missing data on (February 1 through March 3, 2016) and (June 24 through August 9, 2016). This loss of data was caused by the battery on the PRHTemp101A dying and it taking longer than expected to acquire a replacement due to the unusual size of the battery.

## Declaration of Competing Interest

The authors declare that they have no known competing financial interests or personal relationships which have, or could be perceived to have, influenced the work reported in this article.

## Appendix Supplementary materials

Image, application 1
